# IoT in Radiology: Using Raspberry Pi to Automatically Log Telephone Calls in the Reading Room

**DOI:** 10.1007/s10278-018-0081-z

**Published:** 2018-05-03

**Authors:** Po-Hao Chen, Nathan Cross

**Affiliations:** 1 Department of Radiology, Perelman School of Medicine, University of Pennsylvania, 3400 Spruce Street, Philadelphia, PA 19104 USA; 2 Musculoskeletal Imaging, Department of Radiology, Hospital of the University of Pennsylvania, 3737 Market Street, Mailbox #4, Philadelphia, PA 19104 USA; 3 Department of Radiology, University of Washington, 1959 NE Pacific St, Box 357115, Seattle, WA 98195 USA

**Keywords:** Internet of Things, Open hardware, Raspberry pi, Single-board computer

## Abstract

The work environment for medical imaging such as distractions, ergonomics, distance, temperature, humidity, and lighting conditions generates a paucity of data and is difficult to analyze. The emergence of Internet of Things (IoT) with decreasing cost of single-board computers like Raspberry Pi makes creating customized hardware to collect data from the clinical environment within the reach of a clinical imaging informaticist. This article will walk the reader through a series of basic project using a variety sensors and devices in conjunction with a Pi to gather data, culminating in a complex example designed to automatically detect and log telephone calls.

**Electronic supplementary material:**

The online version of this article (10.1007/s10278-018-0081-z) contains supplementary material, which is available to authorized users.

“People who are really serious about software should make their own hardware.”

*– Alan Kay, 20 July 1982*

## Background

Imaging informatics is undergoing a dramatic transformation as the volume, variety, and the velocity of medical text and imaging data have dramatically increased. Tools for analysis are accelerating as well. However, despite the abundance of data in some areas, other processes in the radiology department such as distractions, ergonomics, distance, temperature, humidity, and lighting conditions generate a paucity of data and are difficult to analyze.

The decreasing cost of microcontrollers and single-board computers (SBC) have dramatically decreased the cost of both commercial and custom-made “smart” devices [1]. Among the most successful was Raspberry Pi (RPi). Created by Eben Upton, the RPi series was initially designed for education, but its applicability in other industries and use cases quickly became evident by its commercial success [2]. Upton co-founded Raspberry Pi Foundation for the continuing development of RPi, which also publishes the Magpi magazine (https://www.raspberrypi.org/magpi/).

Despite the popularity of RPi among the open-source community, a paucity of literature exists to describe its potential uses in imaging informatics. We will explore the role of microcontrollers and SBCs in radiology in a separate Journal of Digital Imaging article. In this article, we use the RPi as a primer for imaging informaticists to integrate low-cost sensors to monitor radiology patient care areas and the reading room alike. Then, we demonstrate a framework for connecting many such devices as part of an Internet of Things (IoT) publisher-subscriber architecture.

## Telephone Logger Example

This article will walk you through a project where you use a variety of sensors and devices in conjunction with an RPi to gather data about phone calls.

Telephone logs are readily available in a health system, and phone interruptions have been linked to increased discrepancy [3]. However, the sheer number of calls requiring monitoring limits the granularity of telephone interruptions in such studies to the number, length, and source of incoming calls.

In this telephone log device, a magnet can be secured to the telephone receiver and would close the reed switch when the receiver is in the cradle. Therefore, the position of the handset can be dynamically detected by the change in magnetic force on the reed switch. The goal was to collect information about the content of the phone call while imposing a minimum of work on the user. When many such devices are deployed across many workstations, the data can be published to an IoT server and reviewed by a subscribing dashboard.

We break the project down into small tasks that use different pins on the Raspberry Pi to fulfill specific tasks necessary for the final project. The two rows of GPIO pins on the board all have specific functions which are detailed on a pinout [4]. As you complete one section, leave it assembled when you start on the next and use a different region of your solderless breadboard. That way at the end, the hardware all remains connected, and you just need to alter the code. When considering using this outside of a workshop, you would finalize your design and then have a circuit board made where you could have the components permanently soldered down and made more compact.

The components needed for each section are listed in a bill of materials (BOM) at the start of each section. Some of the parts are generic and could be purchased from a variety of retailers and could be purchased for less. All source code is available under an open source license and provided on GitHub (http://bit.ly/2piVyPz). Alternatively, the source code is also available as Supplementary Material for this article.

### Raspberry Pi Setup

The Raspberry Pi Foundation was founded in 2009 with the goal of promoting the academic study of basic computer science. The Raspberry Pi 3 Model B (RPi3) is the most recent hardware iteration, released in February 2016. The hardware of RPi3 includes a system-on-a-chip (SoC) which integrates several key system components onto the same chip to reduce cost and physical size, including a quad-core ARM processor, 1GB of memory (RAM), and a graphics processing unit (GPU).

An RPi is a blank slate without an operating system until one is flashed onto a microSD card which is inserted on the undersurface of the device. As discussed above, there are a variety of operating systems that can be installed on the RPi. Download and follow the official installation instructions for Raspbian with Desktop: https://www.raspberrypi.org/downloads/raspbian/.

After flashing the operating system to a MicroSD card, connect the RPi3 to HDMI, a keyboard and mouse, and Ethernet if available. Connect the micro USB power supply to the connector on the board. You should start to see indicators light on the edge of the board. With time you should see the boot process scroll by on the monitor, and the OS will walk you through the initial setup.

Once your initial setup has been completed and you have reached the Raspbian desktop, load a terminal and update the package manager ‘apt’ (a package manager is a program which downloads a list of available software packages and will install and uninstall them for you with a few commands):

- $ sudo apt update

This article will primarily use Python to interface and the gpiozero API to interface with attached hardware sensors. Therefore, have ‘apt’ upgrade the existing packages and install new software using the following commands:

- $ sudo apt upgrade
- $ sudo apt install python-dev python-imaging python-smbus python-rpi.gpio build-essential python-gpiozero

These packages include the code to allow python to interact with the GPIO interfaces through two different libraries (rpi.GPIO and gpiozero) and some supporting libraries (Table 1).

**Table 1:** Setup bill of materials

Item	Model	Qty	Price estimate	Adafruit #	Sparkfun #
Raspberry Pi 3 model B	Version 3 model B	1	$40.00	3055	DEV-13825
micro USB Power Supply^a^	500 mA+				
microSD card^a^	<generic> > 8 Gb	1	> $6		
microSD card Reader^a^					

^a^Can be purchased as part of a kit

### LED Example

**Fig. 1:**
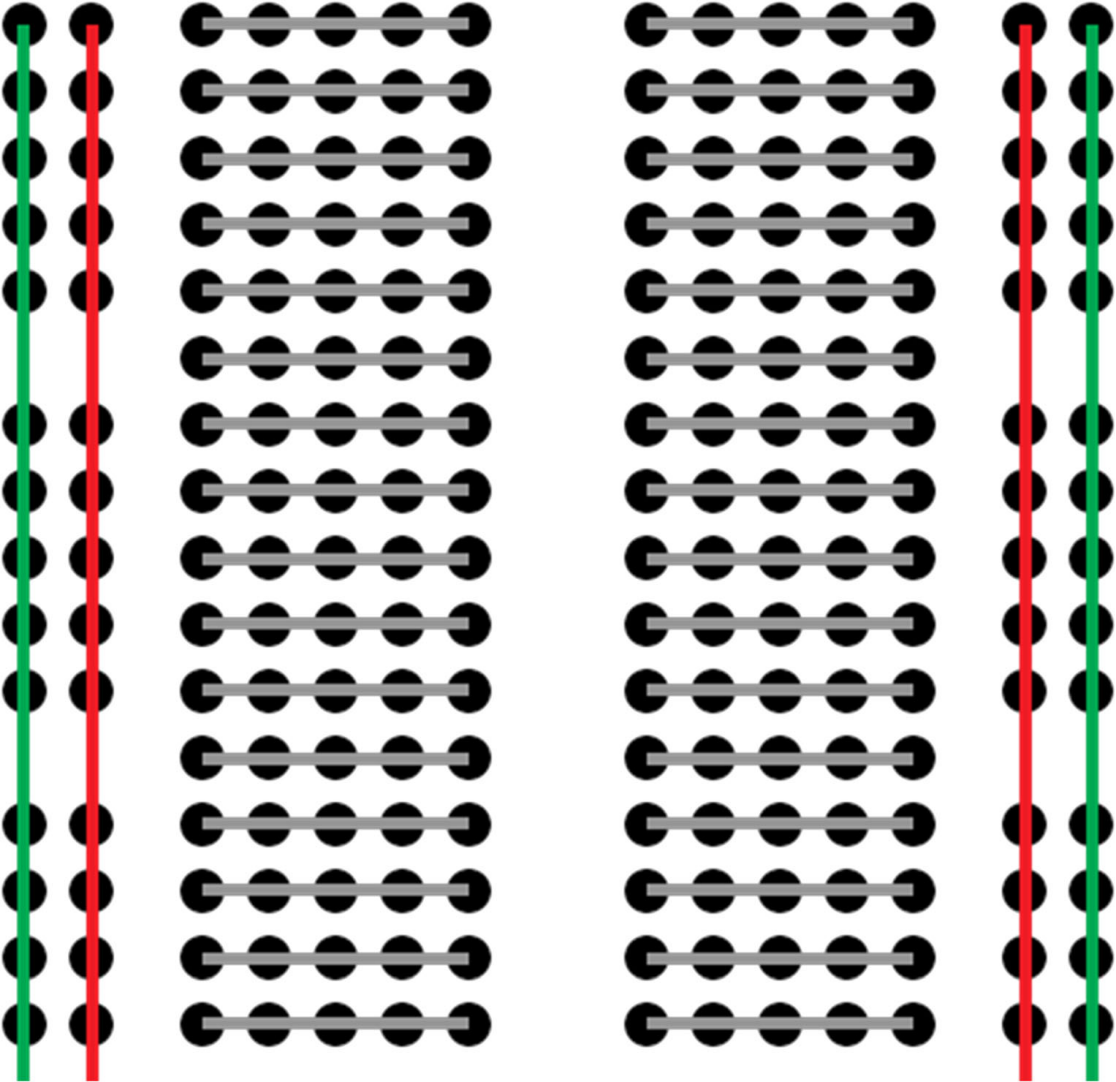
Solderless breadboards. This diagram shows the typical wiring of the pins of a breadboard. Understanding that the five pin holes on either side of the median in the middle of the board are wired together on each side. However, the left side is not wired to the right side. Components are often arranged in the middle of the breadboard with leads going into holes on either side of the median. The two columns of holes on the far edges of the boards are usually used for power supply and ground, and each column is wired as a whole unit

Now that you have a working current operating system installed, you can start interfacing with devices. The bill of materials for this section is shown above in Table 2. It is recommended that whenever you are attaching new devices to the GPIO pins on the RPi3 that you power down the device before connecting. A solderless breadboard is a useful tool for creating circuits quickly. To use one, you must first understand how the pins are interconnected, shown in Fig. 1.

**Table 2:** LED example bill of materials

Item	Model	Qty	Price Estimate	Adafruit #	Sparkfun #
LED	<generic, any color>	2	$0.75	299	COM-09856
Resistords 220 Ω (LED current limiting)	<generic>	2	$0.75	2780	COM-11507^a^
Solderless breadboard	<generic>	1			
female to female jumper wire	<generic>				
male to male jumper wire	<generic>				

^a^Product slightly different but compatible with instruction (resistor value is similar)

The pinout of the RPi3 is a useful reference [4]. Some of the pins on the 2 × 20 header are labeled “+3v3 Power” or “+5v power” meaning that these are pins to supply power to devices but, if shorted to ground, could exceed designed power output and overheat. Since all pins in the two rows look the same, board designers and manufacturers usually mark pin 1 with a small circle. In RPi3, a very small rounded corner of the white silkscreen outline on the green board around the header pins denotes pin 1.

**Fig. 2:**
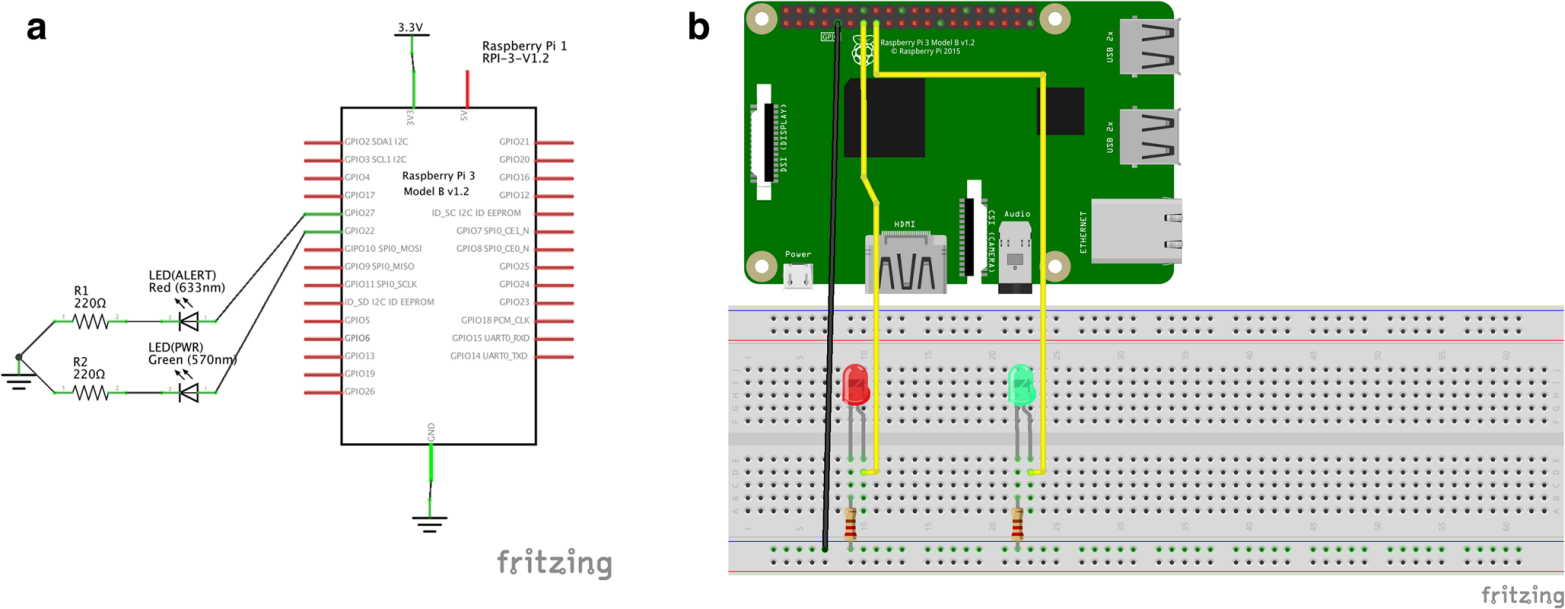
LED example. In this example, two LEDs are wired to different GPIO pins and can be controlled independently in both **a** schematic and **b** breadboard connections

Use the electrical schematic and board model in Fig. 2 to connect two LED (light emitting diodes) of any color to the RPi3’s pins (GPIO22 and GPIO27). Resisters are connected in series with the LEDs to limit the amount of current the LED can draw. The maximum current could be calculated with Eq. 1, where the voltage the RPi3 can supply is + 3.3v and the resistor used is 220 Ω, resulting in a maximal current of 15 mA.

#### Equation 1—Ohm’s Law

$$ V= iR $$

Ohm’s law is one of the fundamental equations for electrical engineering describing the relationship of voltage and current across an area of resistance. V = voltage, i = current, R = resistance.

With the code for this article downloaded, run the python script.

- $ python RadIOT_1_LED.py

This should result in the LEDs lighting in series, 1, then 2, then both, then both off. The gpiozero library documentation describes LED-related functions in more detail [5].

### OLED Display Example

**Fig. 3:**
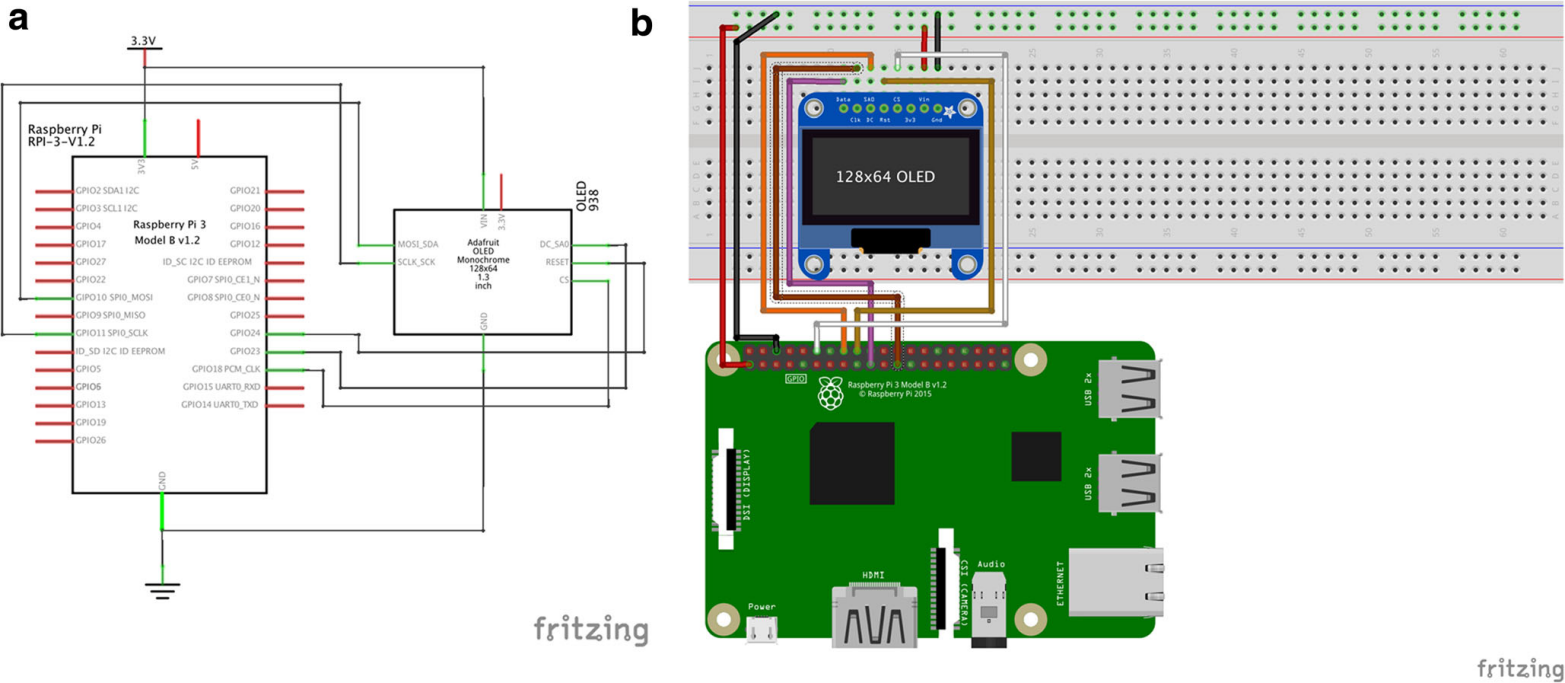
OLED display example. An LCD allows a program to directly output messages to the user from hardware. Using an OLED display requires some different purpose specific pins such as a clock, data, and chip select pin. **a** Schematic of wiring an organic LED device onto RPi3 and **b** the corresponding breadboard connections

Displays are ubiquitous in many electronic devices today. The display in this project uses a common serial communication protocol called SPI (serial peripheral interface) which uses different pins for a clock signal, data, etc. (Table 3). It uses an open source library by Adafruit to simplify the process of communicating with SPI devices. To begin, mount the display on the breadboard and connect the pins from the breadboard to the RPi3 as seen in Fig. 3.

**Table 3:** OLED example bill of materials

Item	Model	Qty	Price estimate	Adafruit #	Sparkfun #
MONOCHROME OLED	1.3″ 128 × 64 px OLED	1	$19.95	938	–

Once the display is connected, execute the commands below.

- $ sudo apt install build-essential python-rpi.gpio python-dev python-imaging python-smbus python-pip git
- $ git clone https://github.com/adafruit/Adafruit_Python_SSD1306.git
- $ cd Adafruit_Python_SSD1306
- $ sudo python setup.py install
- $ sudo raspi-config

The last command will load a menu which allows you to configure many of the settings of your RPi. Use the arrow keys and enter/return key to select “Interfacing Options” > “SPI” > “Yes.” Hit Enter to acknowledge “Ok” and returning to the main menu. Hit tab, right arrow, and enter/return to select “Finish.” The RPi is now setup for serial communication and has the Adafruit SSD1306 library which contains instructions for the SSD1306 chip on the display.

Change directories to the folder containing the code for this lesson. Ensure that the file is executable and the python program which will output a message to the display. Open the python file to understand how the python program controls the display.

- $ python RadIOT_2_OLED.py
- $ nano RadIOT_2_OLED.py

### Button Example

**Fig. 4:**
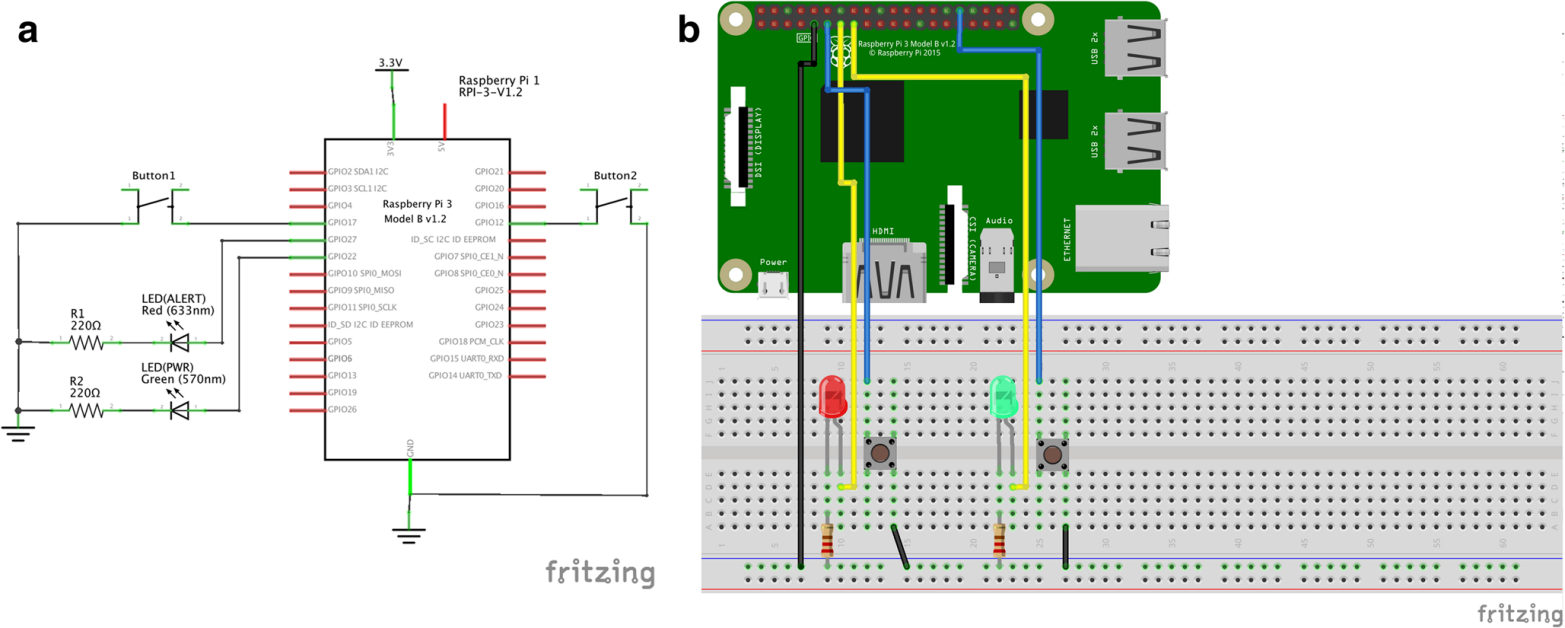
Button example. **a** Schematic of wiring buttons to the LED and **b** the corresponding breadboard connections. Building on the previous LED example, two buttons are added which allow the user to interact with the hardware. In this example, after pressing a button, the code will illuminate the appropriate LED and output a message on the command line

This example illustrates the concept of polling a device to determine its state. A simple tactile pushbutton is connected to a pin on the RPi3, and the opposite side of the pushbutton is connected to ground (Table 4). Internally, a pull-up resistor ensures that when the pushbutton circuit is open, the pin on the RPi is tied to + 3.3 V. When the button is depressed, the pin is pulled to ground. The gpiozero library contains many functions for monitoring the state of a pin including the is_pressed method. Use the wiring diagrams in Fig. 4 to connect the push buttons.

**Table 4:** Button example bill of materials

Item	Model	Qty	Price estimate	Adafruit #	Sparkfun #
Tactile button switch	Tactile button switches	2	~ $2.00	367	COM-00097*

Change directories to the folder containing the code for this lesson. Ensure that the file is executable and run the python program which will light the LED when a button is pressed. Open the python file to understand how the python program polls the pushbuttons and then illuminates the appropriate LED.

- $ python RadIOT_3_Button.py
- $ nano RadIOT_3_Button.py

### Reed Switch Example

A reed switch is a simple device with two parallel metal plates that are pulled together by any adjacent magnet (Table 5). These switches are used in a wide variety of consumer-electronics. In this case, the switch will be used to monitor whether a handset for a telephone is in the cradle or not. The magnet attached to the handset will be pulled away from the reed switch, opening the circuit, when the phone is picked up.

**Table 5:** Reed switch example bill of materials

Item	Model	Qty	Price estimate	Adafruit #	Sparkfun #
Reed switch	Reed switch	1	~ $2.00	375	COM-08642^a^
Magnet	Any household magnet	1	~ $1.00	9	COM-08643^a^

^a^Product slightly different but compatible with instruction (resistor value is similar)

**Fig. 5:**
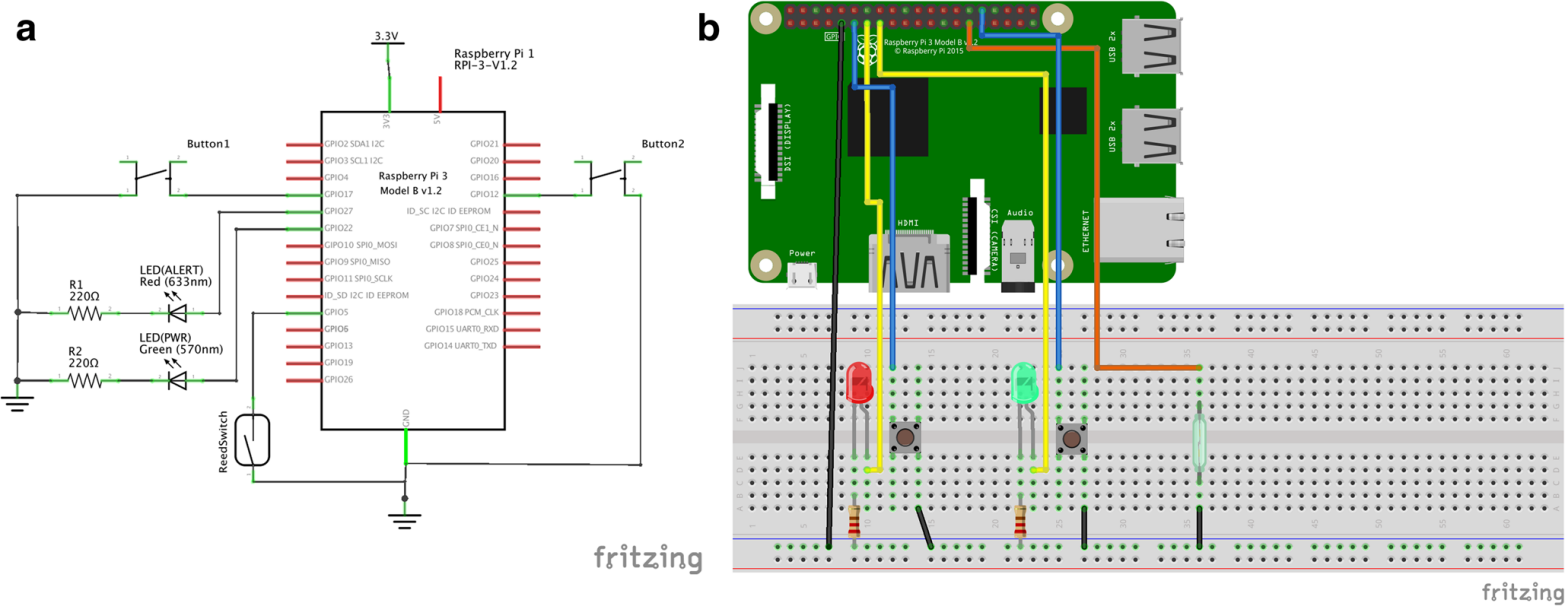
Reed switch example. **a** Schematic of wiring a reed switch to RPi3 and **b** the corresponding breadboard connections. A reed switch is a simple switch used in security systems, door sensors, and motors. The close proximity of a magnet pushes or pulls thin plates of metal together within the glass envelope. In this example, placing a magnet near the reed switch will close the circuit and the program will cause the LEDs to light

Connect to the reed switch as demonstrated in the diagrams below in Fig. 5.

Change directories to the folder containing the code for this lesson. Ensure that the file is executable and run the python program which will poll the state of the reed switch and light the LEDs. Open the python file to understand how the python program polls the reed switch similarly to the pushbutton and then illuminates the appropriate LEDs. A debounce value is used for the reed switch since as the switch closes or opens, there may be a very short period where the connection is intermittently disconnected and connected again. Debouncing is often a good practice on any button or switch.

- $ python RadIOT_4_ReedSwitch.py
- $ nano RadIOT_4_ReedSwitch.py

### Telephone Logging Device

**Fig. 6:**
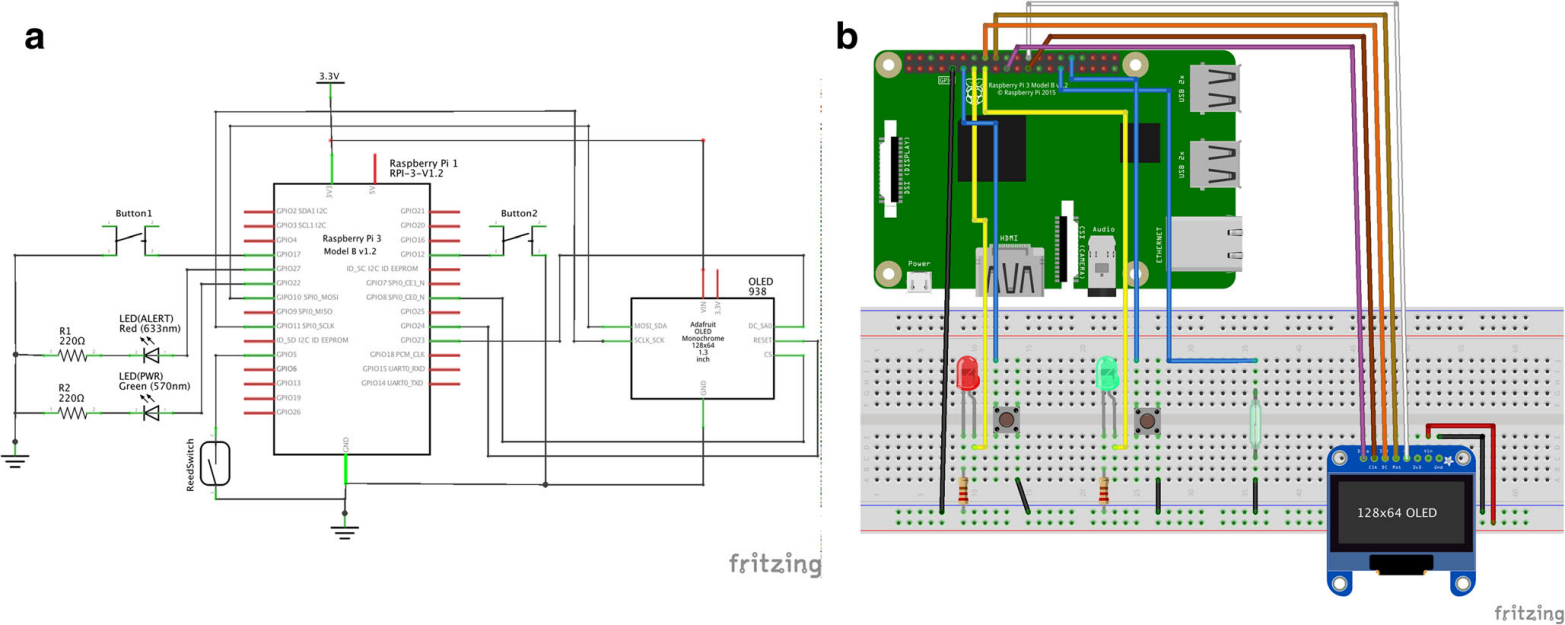
Final cumulative example. **a** Schematic and **b** the corresponding breadboard connections of wiring LEDs, buttons, a OLED display, and reed switch to RPi3 to create a telephone call logging device for the radiology reading room. If a magnet is glued to the receiver and the reed switch is placed on the phone stand, replacing the receiver on the stand will be detected by the program which will light up the LEDs to get the attention of the user. A question will appear on the screen, and the user can press a button next to an appropriate answer. The LED next to the button will light to give the user feedback that the answer was recorded

In this example, all of the previously connected devices are used together to create a more complex system (Fig. 6). The code is designed to monitor the reed switch till the handset is picked up opening the reed switch circuit. When the reed switch circuit is closed again, the program outputs a message to the OLED display requesting the user to press one of the two buttons corresponding to the answer which will light up an LED in confirmation that the message was recorded. Also, confirmation is displayed on the OLED display. The code records the response and timestamp to a database file for further analysis later.

To execute the code change to the directory for the exercise, ensure that the file is executable and run the Python program. Then, review the code and comments in your editor of choice.

- $ python RadIOT_5_Cummulative.py
- $ nano RadIOT_5_Cummulative.py

### Data Storage and Communication

So far, the device has no capability of storing or communicating the data it has collected. There are several ways of keeping the data selection. First, the data may be stored locally on the RPi3 flash disk, but this approach becomes quickly unmanageable with increasing number of devices. The data may be stored in a network database for processing later, but this process may be too cumbersome for real-time dashboards. Instead, the data may be published through a dedicated network protocol to a lightweight message hub so that databases, real-time dashboards, and other resources may subscribe to the data stream and perform their own respective downstream processing.

**Fig. 7:**
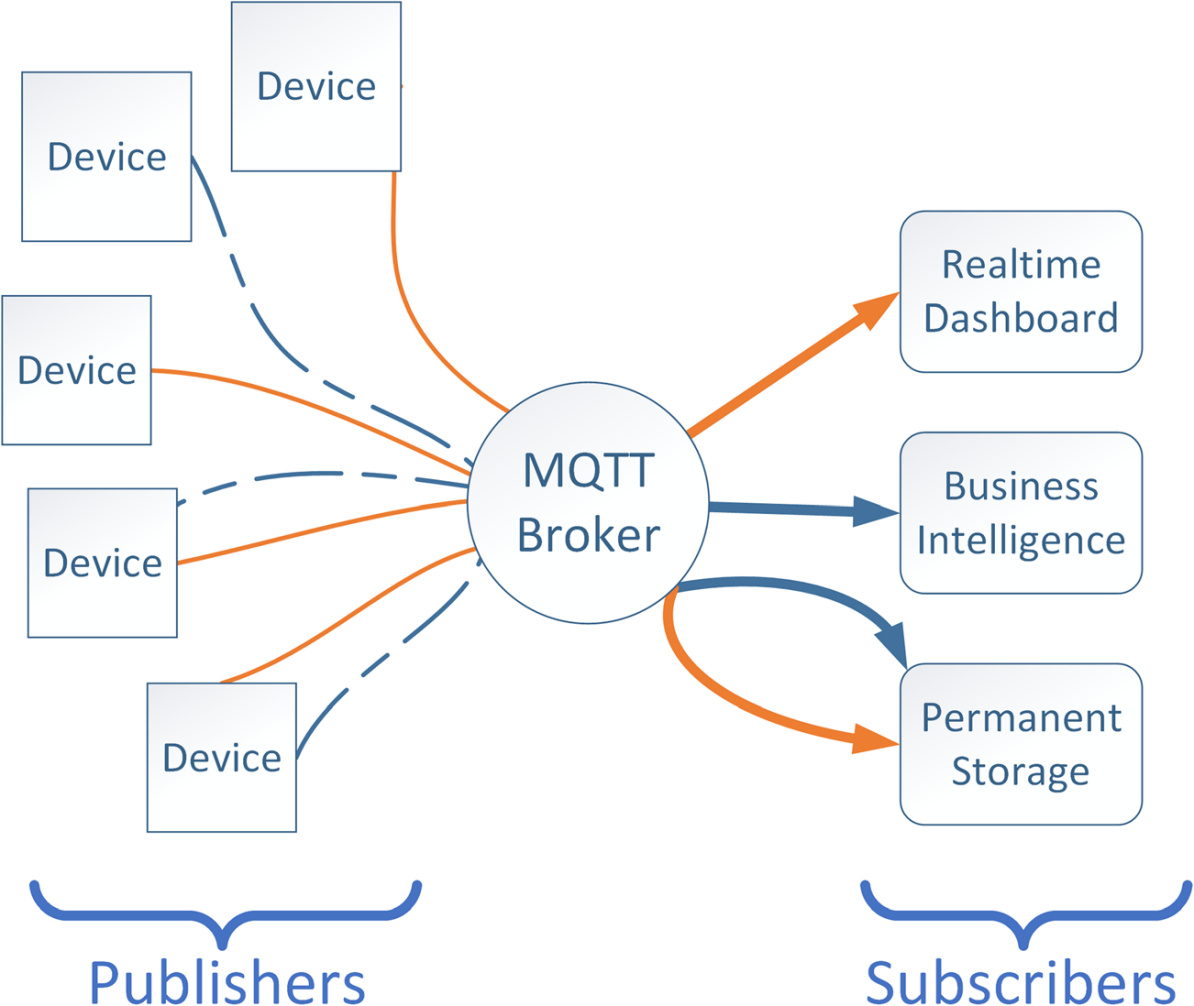
Publisher-subscriber communication. A device may publish on any number of “topics,” denoted by solid and dotted lines. Subscribers may choose to consume any or all topics even without detailed knowledge of the data publishers

Message Queue Telemetry Transport (MQTT) is a lightweight, popular IoT messaging protocol communicating under TCP/IP through the publish-subscribe pattern of communication (Fig. 7). In these communication patterns, IoT devices collect and categorize data by topic but do not specifically address a receiver when sending. Instead, the devices “publish” data to a central resource. Users wishing to consume the data may “subscribe” to specific topics and receive push notifications when data has been published on the topic of choice.

- $ pip install paho-mqtt

The code in RadIOT_6_SendData.py adds basic MQTT capabilities to the RPi3. An example subscriber is included under RadIOT_DBSubscriber.py which may be run on any network-connected computer running Python, not necessarily a Raspberry Pi. The topic and location variables need to be properly configured to ensure the publishers and subscribers are addressing the same topics.

$ python RadIOT_6_SendData.py.

$ nano RadIOT_6_SendData.py.

## Conclusions

In this article, we combine hardware sensors with an RPi3 and demonstrate an automatic telephone log device capable of capturing environmental events both automatically and by user-input. Then, we connected the device to an MQTT broker as a data publisher. The cost and threshold for developing customized sensors and hardware is low. Customized hardware and sensors have immense potential for the radiology department, providing data beyond that which imaging informaticists traditionally have access.

## Electronic supplementary material

ESM 1 (PY 1 kb)

ESM 2 (PY 1 kb)

ESM 3 (PY 1 kb)

ESM 4 (PY 1 kb)

ESM 5 (PY 1 kb)

ESM 6 (PY 4 kb)

ESM 7 (PY 5 kb)

ESM 8 (PY 3 kb)
